# Metabolic imprinting in beef calves supplemented with creep feeding on performance, reproductive efficiency and metabolome profile

**DOI:** 10.1038/s41598-024-60216-1

**Published:** 2024-04-27

**Authors:** Bruna Lima Chechin Catussi, Jaqueline Rodrigues Ferreira, Edson Guimarães Lo Turco, Sérgio Carlos Franco Morgulis, Pietro Sampaio Baruselli

**Affiliations:** 1 Department of Animal Reproduction, School of Veterinary Medicine and Animal Science, University of São Paulo, São Paulo, SP Brazil; 2 Federal University of Mato grosso do Sul, Campo Grande, MS Brazil; 3 Ion Medicine Clinical Metabolomics, São Paulo, SP Brazil; 4 Minerthal Nutritional Products, São Paulo, SP Brazil

**Keywords:** Biotechnology, Animal biotechnology, Metabolomics

## Abstract

This experiment evaluated the influence of creep feeding supplementation on productive and reproductive performance and on serum metabolome profile in Nelore (*Bos indicus*) heifers. Female calves were assigned to treatments: Creep (n = 190), with ad libitum access to a nutritional supplement from 70 to 220 days after birth, or Control (n = 140), without supplementation. After weaning (Day 220), both groups followed the same pasture and nutritional management. Body weight (BW) and backfat thickness (BFAT) were measured over time. Blood samples were collected at 220 and 360 days for LC–MS/MS targeted metabolomics. On day 408, during the synchronization timed artificial insemination (TAI) protocol, reproductive status (RS: diameter of uterine horn and largest follicle, and presence of CL) was assessed. Creep feeding increased BW and BFAT at weaning, but no differences in BW, BFAT, or RS after weaning were observed. Nonetheless, the pregnancy per AI (P/AI) for 1st service was 28.9% higher in the Creep group. On day 220, 11 significant metabolites influenced five metabolic pathways: Glucose-alanine cycle, alanine, glutathione, phenylalanine and tyrosine metabolism, and urea cycle. On day 360, 14 significant metabolites influenced eight metabolic pathways: Malate-aspartate shuttle, arginine and proline metabolism, urea cycle, aspartate, beta-alanine, glutamate metabolism, ammonia recycling and citric acid cycle. In conclusion, creep feeding supplementation improved calf performance and induced metabolic changes at weaning and 360 days of age. Although heifers had similar productive performance and reproductive status, when submitted to TAI, those supplemented with creep feeding had greater P/AI.

## Introduction

Enhancing productivity and reproductive efficiency in beef cattle herds is crucial for sustainable livestock intensification programs^1,2^. Particularly in Bos indicus cattle, achieving puberty at older ages (around 22–36 months) represents a significant challenge for improving beef production efficiency^3^. Consequently, the late first calving of heifers not only impairs the implementation of intensive beef production practices but also contributes to adverse environmental impacts, such as greenhouse gas emissions (GHG)^4,5^. Studies have demonstrated that reducing the age at first calving and implementing strategies to increase fertility can substantially reduce GHG emissions (25–37%), promoting greater economic returns over the entire beef production cycle^6,7^. Therefore, understanding the mechanisms that impact the performance and fertility of young beef heifers can significantly enhance the productivity and sustainability of beef production systems.

The onset of puberty in heifers is strongly influenced by nutritional status and body development, crucial in signaling sexual maturity^8–10^. Adequate nutrition and optimal body development are prerequisites for attenuating estradiol negative feedback and releasing of luteinizing hormone (LH)^11^. Leptin, primarily secreted by adipocytes, notably influences puberty initiation^12^. Acting on Kiss1 neurons, leptin likely mediates positive impacts on GnRH and LH release in ruminants through intermediate pathways. In addition, other hormones (insulin and ghrelin) and nutrients (glucose, fatty acids, and amino acids) contribute to a neural network sensing metabolic fuel availability, ultimately influencing reproductive functions^9^. Hence, modulating the growth rate and, particularly, body composition of calves by nutritional strategies during the suckling phase may alter metabolic pathways during early life, posteriorly affecting health and performance parameters due to the metabolic imprinting effect^13,14^. The term metabolic imprinting defines an epigenetic change in response to a nutritional intervention during early life, which permanently alters physiological outcomes in later life^15,16^. Critical windows of organ development extend into the postnatal period, during which specific regulatory mechanisms, such as the development of pancreatic islets and neurons, continue maturing in rodents^17^. Thus, a modified nutritional experience during the early-life phase may stimulate metabolic imprinting, impacting these crucial developmental stages.

This epigenetic phenomenon spans several species, including mice, humans, pigs, as well as dairy and beef cattle. In rats, undernourished altered neuronal responses in various hypothalamic centers in response to insulin, leptin, and neuropeptides^18^. Nutrient restriction in piglets hindered skeletal muscle growth and delayed muscle fiber maturation^19^. Moreover, studies in dairy cattle demonstrated the positive impact of increased preweaning nutrient intake on subsequent lactation performance compared to calves subjected to restricted feeding^20^. In beef cattle, creep feeding in nursing calves enhanced body weight (BW) gain^21–23^ and reduced age at puberty in heifers^11,24,25^. Cardoso et al.^26^ proposed that improved nutrition in heifers relates to reduced neuropeptide Y (NPY) mRNA expression per neuron compared to low-nutrition heifers. Furthermore, heifers under improved nutrition exhibited increased proopiomelanocortin (POMC) mRNA expression and greater density per cell in the arcuate nucleus. Moreover, increased melanocyte-stimulating hormone α (αMSH) connections to kisspeptin/neurokinin dynorphin (KNDy) neurons were observed. These findings collectively suggest that enhanced juvenile nutrition elevates metabolic hormone levels, influencing gene expression and neuronal projections, ultimately impacting KNDy and GnRH neurons^27^.

Therefore, epigenetic mechanisms appear to be implicated; an increase in nutritional levels between 4 and 8 months altered gene methylation in the arcuate nucleus linked to regulating growth and development^28^. However, the metabolic mechanisms involved with epigenetics are still unclear. Thus, the most recent omics technology, metabolomics, uses innovative analytical chemistry techniques to measure large numbers of metabolites in several biological matrices, such as serum blood^29^. Metabolomics, as a post-genomics tool, provides clear information about the metabolic status of an organism, offering a promising opportunity to elucidate the influence of nutritional management on metabolic and physiological outcomes^29,30^. Thus, the aim of this study was to evaluate the effect of creep feeding supplementation during the preweaning phase on productive and reproductive performance, as well as on the serum metabolome profile of young beef heifers. In the context of optimizing productivity and reproductive performance in beef farms, we hypothesized that supplementing female calves by creep feeding would improve performance and induce alterations in their metabolic profile during early life, posteriorly resulting in improved metabolism and reproductive performance in the first breeding season.

## Material and methods

### Experimental design

The present study was conducted following ethical directives for animal experiments, complied with ARRIVE guidelines^31^, and was approved by the ethical committee in using animals of the School of Veterinary Medicine and Animal Science of University of São Paulo (CEUA-FMVZ/USP, No. 7317190623). All methods were performed in accordance with the relevant guidelines and regulations. A total of 330 Nelore (Bos indicus) suckling female calves (with their respective dams) from a commercial beef farm (Santa Rita do Pardo, Mato Grosso do Sul, Brazil) were randomized by calving date and randomly allocated in five pastures (Brachiaria decumbens) with free-choice access to water and mineral supplements. The calves’ birth date was considered Day 0 of the experiment. At the beginning of the supplementation period, calves were 70.1 ± 2.8 days old (mean ± SEM), and the mean BW was 113.5 ± 3.2 kg. Calves were designated to receive one of two nutritional treatments, according to the pasture where they were allocated: (1) Control = no calf supplementation (2 pastures; 70 cow-calf per pasture); or (2) Creep: creep feeding supplementation (3 pastures; 63 cow-calf per pasture). The supplement was provided in cow exclusion areas and consisted of molasses blocks (MB) formulations (Minerblock^®^ Creep, Minerthal Nutritional products, São Paulo, Brazil; Table 1) in the proportion of 1 block (25 kg) per each 20 calves. Calves had continuous and free-choice access to MB until the weaning. MB consumption was estimated by the weight reduction of each block, measured weekly, and replaced when the block weight was < 3 kg. The creep feeding supplementation was provided for 150 days, from Day 70 to Day 220. Supplement intake of each pasture was divided by the number of calves within each pasture and presented as grams per calf/day.

**Table 1:** Average chemical composition of creep feeding supplement provided from day 70 to 220 and post weaning supplement provided from Day 221 to 458.

Item^b^	Supplement^a^	
	Creep feeding	Post-weaning
TDN, %	62.5%	67.0%
CP, %	20.0%	20.0%
Ca, g/kg	45.0	20.0
P, g/kg	7.5	10.0
Na, g/kg	15.0	65.0
Zn, mg/kg	360.0	400.0
Cu, mg/kg	120.0	155.0
I, mg/kg	6.0	14.0
Co, mg/kg	7.5	10.0
Se, mg/kg	3.0	7.0
Mn, mg/kg	202.5	–
Virginiamycin, mg/kg	125.0	–

^a^Creep feeding = Molasses block supplementation offered ad libitum during the pre-weaning; Post weaning = Supplementation offered at 0.3% of body weight/animal/day.

^b^*TDN* total digestible nutrients, *CP* crude protein, *Ca* calcium, *P* phosphorous, *Na* sodium, *Zn* zinc, *Cu* copper, *I* iodine, *Co* cobalt, *Se* selenium, *Mn* manganese.

On day 220, all calves/heifers were weaned and allocated to a single pasture (70 ha; Brachiaria decumbens), where they remained during the postweaning period. They had free-choice access to water and received an energetic-protein supplement (PSAI PP14 Fator P; Premix, Ribeirão Preto, São Paulo, Brazil) provided at 0.3% of BW until the end of the experiment (Day 456). The chemical composition of the creep feeding and post-weaning supplementation are shown in Table 1.

### Reproductive management

Initially, all heifers were submitted to a cyclicity induction protocol, receiving 150 mg of long-acting P4 i.m. (Sincrogest, Ourofino, Cravinhos, São Paulo, Brasil) on day 360, and a second dose 24 days apart on Day 384. The timed artificial insemination (TAI) protocol was started 48 days after the beginning of induction protocol (Day 408), with the following protocol: D0: insert of a 0.6 g intravaginal progesterone (P4) device (Fertilcare 600, MSD, São Paulo, Brazil), 2.0 mg of estradiol benzoate i.m. (Fertilcare sincronização, MSD) and 0.530 mg of cloprostenol sodium i.m. (Ciosin, MSD); D7: P4 device withdrawal, 0.530 mg of cloprostenol sodium i.m. (Ciosin, MSD), 0.5 mg of estradiol cypionate i.m. (Fertilcare ovulação, MSD), and 200 IU of equine chorionic gonadotropin i.m. (Folligon, MSD). On D9 of the TAI protocol, all heifers were inseminated by the same technician using one semen batch from one previously tested bull. Pregnancy diagnosis was performed by transrectal ultrasonography (S8, Sonoscape; Domed-Dominium Medical, USA) 30 days after the 1st TAI (Day 447). Non-pregnant heifers were resynchronized to a 2nd TAI following the same hormonal protocol described. Pregnancy diagnosis of the 2nd TAI was performed on Day 486. The cumulative pregnancy rate was determined based on the total number of pregnant heifers from both TAI.

### Data collection

Individual BW was measured on Days 70, 132, 220, 360, and 408 after 12 h of feed and water restriction. The average daily gain (ADG) for the pre-weaning period was calculated based on the BW on Days 70 and 210. The body condition score (BCS) was assessed by a single trained individual on days 360 and 408, using a 1 (very thin) to 5 (very fat) point scale^32^. Moreover, on days 220 and 360, the rump fat thickness (RFAT) was assessed by carcass ultrasonography (Aloka 500 SV; Hitachi Aloka Medical America, CT, USA). RFAT measurements were taken when the transducer was linearly positioned between hooks and pins at the sacral examination site and moved slightly to obtain the correct ultrasound image, allowing for the visualization of the superior limit of the biceps femuris muscles^33,34^. Carcass ultrasound examinations were performed by a single technician, and images were processed using the Lince software (M & S Consultoria Agropecuária Ltda., SP, Brazil).

Evaluation of the reproductive tract^35^ was conducted via transrectal ultrasonography (S8, Sonoscape; Domed-Dominium Medical, USA) on days 360 and 408, including assessment of cyclicity rate based on corpus luteum (CL) presence. Measurements of uterine horn diameter (UT) and largest follicle diameter (LF) were also taken on these days. Both diameters were calculated by taking each structure's mean width and length from a frozen ultrasound image. Blood samples were randomly collected from a subset of heifers (n = 40 per treatment) from the jugular vein on days 220 and 360 to perform metabolomics analyses. All samples were collected on the same day, under consistent conditions, ensuring uniformity in the sampling process. Serum samples, obtained using a tube containing a clot activator (Vacutainer; Becton Dickinson, Franklin Lakes, NJ, USA), were immediately placed on ice and centrifuged at 1200 × g for 30 min for serum recovery, then stored at − 80 °C on the collection day.

### Target metabolomics

Metabolomics analyses were conducted by IonMedicine Clinical Metabolomics (São Paulo, SP, Brazil) utilizing targeted metabolomics analysis to assess specific pre-selected metabolites. Liquid chromatography with triple quadrupole mass spectrometry detection (LC–MS/MS; Shimadzu 8060NX, Kyoto, Japan) was employed in both positive and negative modes (ESI + , ESI-)^36^. Analytical standards and reagents were sourced from Sigma-Aldrich (St. Louis, MO, USA) and Avanti polar Lipids (Birmingham, AL, USA), with isotopically labeled standards acquired from Sigma-Aldrich and Cambridge Isotope Laboratories (Tewksbury, MA, USA), all with purity exceeding 98%. Thawed samples underwent a 50µL aliquoting process into a 96-well deep-well plate, followed by the addition of a 200µL precipitating solution (acetonitrile/IPA/formic acid, 69:30:1 ratio) containing a mix of isotopically labeled internal standards. After homogenization and storage at − 20 °C for 5 min for protein precipitation, samples were centrifuged at 3500 RPM for 15 min. A 20µL aliquot was then resuspended with 200µL of LCMS-grade water for injection. Chromatographic separation utilized two reverse-phase columns: Discovery HS F5-T3 (2.1 mm x 150 mm x 3 µm) for amino acids, nucleotides, and organic acids, and Phenomenex Kinetex C8 (2.1 mm x 150 mm, 2.6 µm; Torrance, CA, USA) for lipids. Collision energies (CE) were optimized automatically for each metabolite. Source conditions included nebulizing gas flow rate: 3.0 L/min, drying gas flow rate: 10 L/min, heating gas flow rate: 10 L/min, interface temperature: 300 °C, DL temperature: 250 °C, block heater temperature: 400 °C, and ionization mode: ESI+/−. Polarity optimization in multiple reaction monitoring (MRM) mode was used for each metabolite. Quantification of metabolite concentration and quality control assessment were conducted using LabSolutions CL and LabSolutions Insight (Shimadzu).

### Statistical analyses

This experiment followed a randomized design, with a random effect of pasture (group of heifers in a pasture in which treatment was applied) nested within treatment^37^. Distributions of the residuals of continuous data, such as BW, ADG, RFAT, UT and LF were evaluated for normality using graphical diagnostics, and when necessary, data transformation was performed, and outliers were removed. Data were analyzed by the GLIMMIX procedure of SAS (SAS Institute Inc., Cary, NC). The covariance structure was determined using the unstructured method. Based on the smallest Akaike’s information criterion values, the best-suited covariance structure was Unstructured UN(1). Other covariance structures were tested, including compound symmetry, heterogeneous compound symmetry, first-order autoregressive, and heterogeneous autoregressive. In addition, the data from the first sampling date of BW on Day 70 and age of heifers were added as a covariate in the statistical model for BW over time.

Continuous data are presented as means ± SEM. Statistical significance was defined as *P* ≤ 0.05. Variables with a binomial distribution (cyclicity rate and P/AI) were analyzed by logistic regression using the GLIMMIX procedure (SAS). Initial models considered the following categorical explanatory variables as fixed effects: treatment, BCS, and their respective interactions. Heifer within the pastures were included as a random effect in the model. The fixed effects model that best fit the data for each variable of interest was selected by finding the model with the lowest value for the Akaike information criterion using a backward elimination procedure that sequentially removed all variables with *P* ≥ 0.10 from the model. The final models included the fixed effects of treatment and the random effect of heifer within the pastures.

In addition, the metabolite concentration table was uploaded to MetaboAnalyst 5.0^38^, and the data transformation included normalization by sum and Pareto scaling. The list of molecules within each biochemical class is in Supplementary Table S1. The supervised partial least squares discriminant analysis (PLS-DA) method was performed for the two periods analyzed (Days 220 and 360). Cross-validation was performed for PLS-DA using the leave-one-group-out cross-validation method (LOOCV), and the performance measure accuracy (Day 220: R2 = 0.87, accuracy = 0.61; Day 360: R2 = 0.78, accuracy = 0.66). The variable importance in the projection (VIP) plot was used to rank the metabolites based on their importance in the treatments. Metabolites with the highest VIP values were the most powerful group discriminators. The VIP values > 1.5 were significant.

## Results

### Growth and body parameters

**Figure 1:**
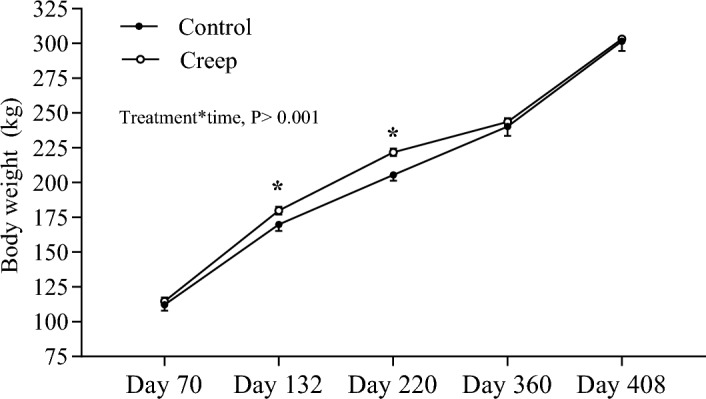
Body weight gain (mean ± SEM) of Nelore heifers according to treatments (Control vs. Creep). The supplementation period was from Day 70 to Day 220. After weaning (Day 220) heifers were kept in the same pasture and under same nutritional management until the end of the experiment. * Means statistically different (*P* < 0.05).

During the creep feeding phase, the average daily intake of MB supplement was 291.9 ± 49.6 g/heifer/day, corresponding to 0.174 ± 0.02% of calf BW based on the average BW during this phase (167.9 ± 1.4 kg). The ADG during the creep feeding was greater in Creep than in the Control group (0.75 ± 0.01 vs. 0.68 ± 0.01 kg/day; *P* = 0.001). The BW over time was affected by treatment, time, and the interaction treatment × time (*P* < 0.001; Fig. 1). At the beginning of the nutritional treatment (Day 70), heifers had the same BW (Creep = 114.7 ± 0.18 vs. Control = 112.1 ± 0.20; *P* = 0.52). On Day 132, heifers from the Creep group were heavier than heifers from the Control group (179.9 ± 1.8 vs. 169.9 ± 2.7 kg; *P* = 0.01). Likewise, at weaning (Day 220), heifers from the Creep group were 10.4 kg heavier than Control heifers (221.9 ± 1.7 kg vs. 205.5 ± 2.7 kg; *P* < 0.001). However, BW did not differ between treatments during the post-weaning period, neither on Day 360 (*P* = 0.53) nor on Day 408 (*P* = 0.95). At weaning (Day 220), heifers supplemented with creep feeding had greater RFAT than control heifers (4.75 ± 0.1 vs. 4.24 ± 0.1 mm; *P* = 0.03). However, no differences between treatments were found in RFAT (*P* = 0.81) on Day 360. Moreover, the BCS on Day 360 (*P* = 0.87) were similar for both groups.

### Reproductive parameters

**Figure 2:**
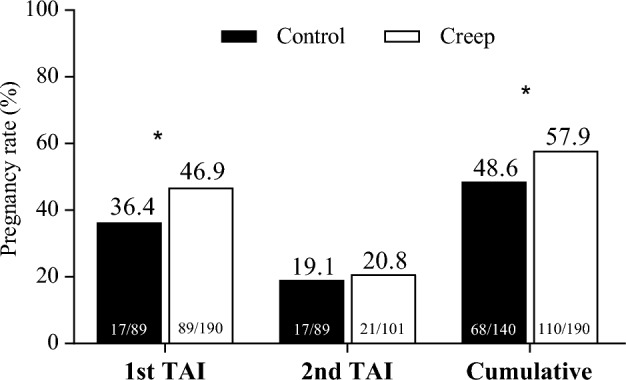
Pregnancy per AI (%) at the first and second TAI, and cumulative pregnancy rate (1st + 2nd TAI) according to treatments (control vs. creep) in young Nelore heifers. * Means statistically differ (*P* < 0.05).

On Day 360, heifers from both treatments had similar UT (*P* = 0.75) and LF (*P* = 0.93; Table 2). In addition, the cyclicity rate did not differ (*P* = 0.98) between treatments on this day. At the onset of the TAI protocol (Day 408), neither LF (*P* = 0.75) or cyclicity (*P* = 0.91) were different between the creep and control groups (Table 2). Despite that, the P/AI after 1st TAI was 10.4 percentage points greater in Creep than in the Control group [46.8 (89/190) vs. 36.4% (51/140); *P* = 0.05], although no effect had been observed on P/AI after 2nd TAI (*P* = 0.69; Fig. 2). Nevertheless, heifers from the Creep group had greater cumulative P/AI when compared with the Control group [57.9 (110/190) vs. 48.6% (64/140); *P* = 0.05].

**Table 2:** Effect of creep feeding supplementation on growth and reproductive parameters in Nelore heifers at different times.

Item^a^	Treatment		*P* value
	Control (n = 140)	Creep (n = 190)	
Day 220			
Body weight, Kg	205.5 ± 2.69	221.9 ± 1.74	0.001
RFAT, mm	4.24 ± 0.09	4.75 ± 0.09	0.03
Day 360			
Body weight, Kg	240.4 ± 2.74	243.6 ± 1.77	0.53
BCS	2.64 ± 0.02	2.65 ± 0.02	0.87
RFAT, mm	2.69 ± 0.07	2.70 ± 0.06	0.81
UT, mm	11.0 ± 0.18	11.0 ± 0.14	0.75
L F, mm	9.68 ± 0.15	9.66 ± 0.12	0.93
Cyclicity rate, % (n/n)	0.7% (1/140)	1.6% (3/190)	0.98
Day 408			
Body weight, Kg	301.5 ± 3.87	303.2 ± 2.37	0.95
BCS	2.99 ± 0.02	3.00 ± 0.02	0.78
LF, mm	10.9 ± 0.16	10.6 ± 0.13	0.75
Cyclicity rate, % (n/n)	28.6% (40/140)	28.9% (55/190)	0.91

^a^*RFAT* subcutaneous rump fat thickness, *BCS* body condition score (1–5 scale), *UT* uterus diameter, *LF* largest follicle diameter.

### Partial least squares discriminant analysis (PLS-DA)

**Figure 3:**
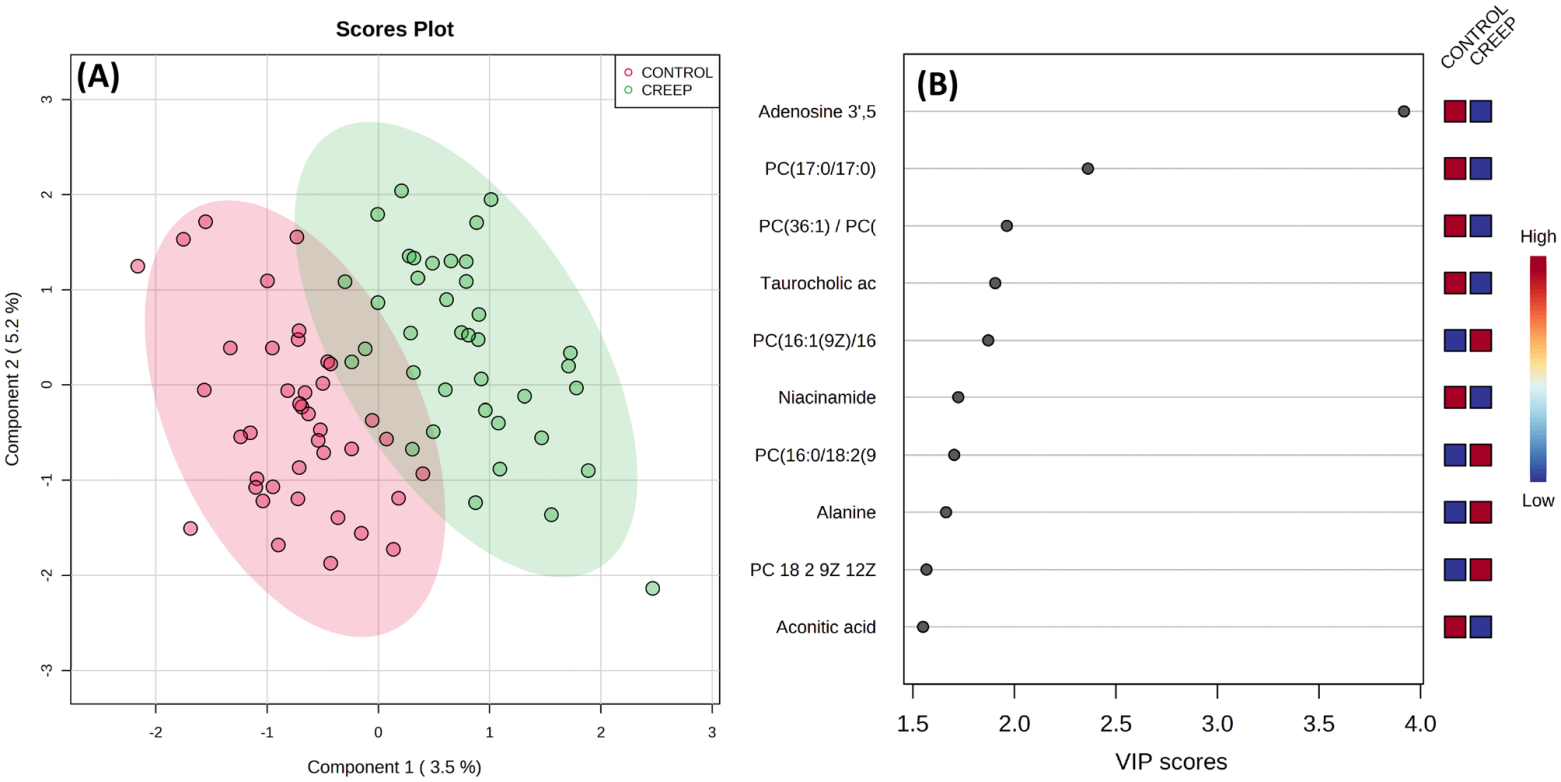
Partial least squares discriminant analysis (PLS-DA) plot of metabolome of serum blood (**A**) and the top metabolites (VIP scores) list (**B**) of Nelore calves on day 220 (weaning date) supplemented (n = 40) or not (n = 40) with creep feeding.

**Figure 4:**
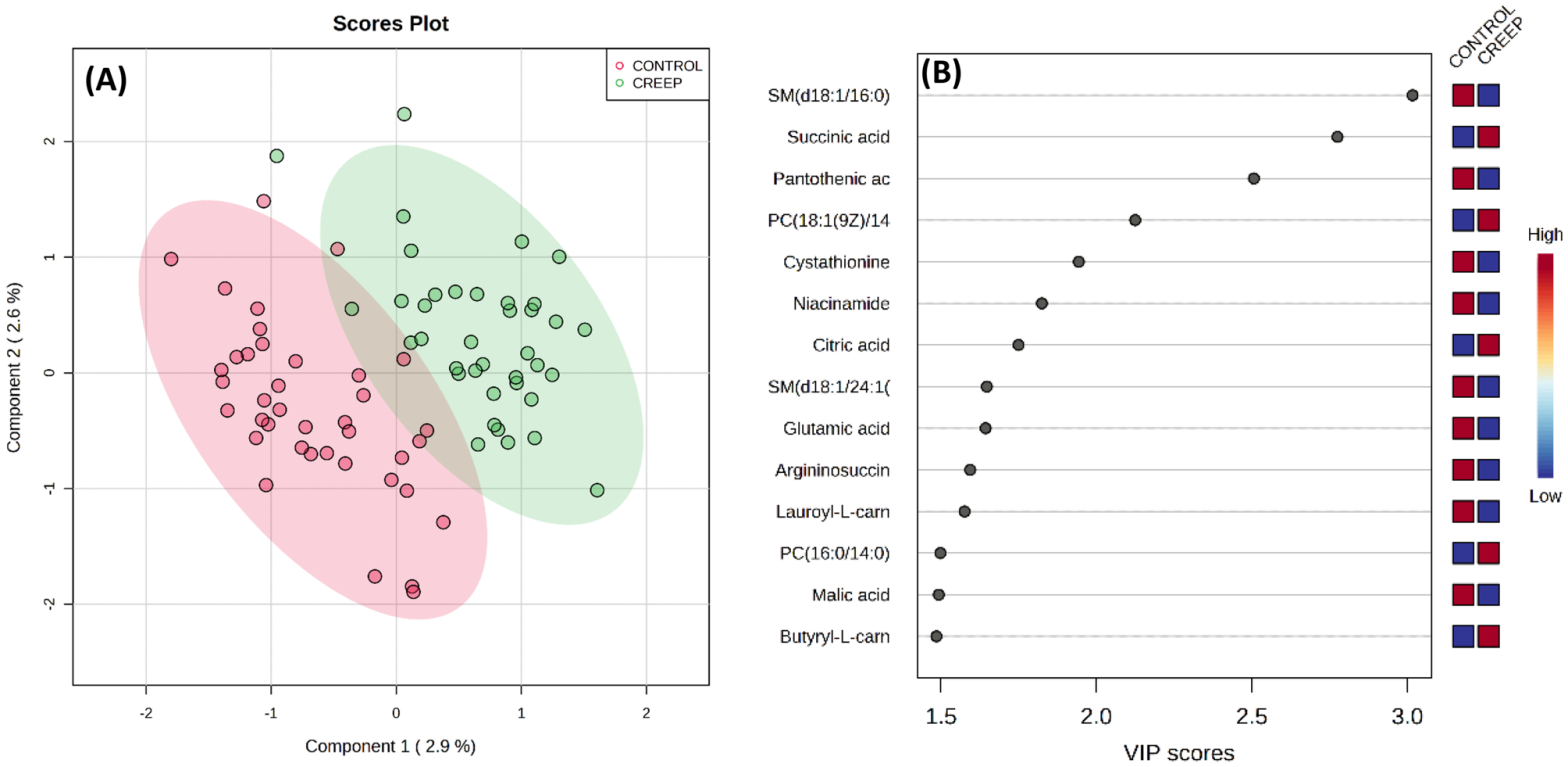
Partial least squares discriminant analysis (PLS-DA) plot of metabolome of serum blood (**A**) and the top metabolites (VIP scores) list (**B**) of Nelore heifers on day 360 supplemented (n = 40) or not (n = 40) with creep feeding.

During this study, 84 metabolites were identified and analyzed. Based on variable importance in the projection (VIP) scores, metabolites that contributed to a higher percentage of the residuals explained in the PLS-DA plot (Fig. 3) between treatments in heifers on Day 220 were: Adenosine 3',5'-cyclic monophosphate (ACM; VIP = 3.919), 1,2-Diheptadecanoyl-sn-glycero-3-phosphorylcholine (PC17:/17:0; VIP = 2.362), PC(16:1(9Z)/16:1(9Z))/ Phosphatidylcholine(32:2) (PC36:1/ PC18:1; VIP = 1.963), taurocholic acid (VIP = 1.906), 1,2-Dinonadecanoyl-sn-glycero-3-phosphocholine (PC 16:1 9Z 16; VIP = 1.871), niacinamide (VIP = 1.724), 1-Palmitoyl-2-linoleoyl-sn-glycero-3-phosphocholine (PC 16:0/18:2; VIP = 1.704), alanine (VIP = 1.664), 1-Linoleyl 2-linoleoyl-sn-glycer-3-phosphocholine (PC 18 2 9Z 12Z; VIP = 1.5672), aconitic acid (VIP = 1.551). Based on variable importance in the VIP scores, compounds that contributed to a higher percentage of the residuals explained in the PLS-DA plot (Fig. 4) between treatments in heifers on day 360 were: N-Palmitoylsphingomyelin (SM d18:1/16:0; VIP = 3.017), succinic acid (VIP = 2.775), pantothenic acid (VIP = 2.507), 1-Oleoyl-2-myristoyl-sn-glycerol-3-phosphocholine (PC 18:1 9Z/14; VIP = 2.125), cystathionine (VIP = 1.944), niacinamide (VIP = 1.825), citric acid (VIP = 1.750), C24:1 Sphingomyelin (SM d18:1/24:1; VIP = 1.648), glutamic acid (VIP = 1.644), argininosuccinic acid (VIP = 1.595), lauroyl-L-carnitine (VIP = 1.577), 1-Palmitoyl-2-myristoyl-sn-glycerol-3-phosphocholine (PC 16:0/14:0; VIP = 1.509), malic acid (VIP = 1.501) and butyryl-L-carnitine (VIP = 1.500).

### Enrichment analysis

**Figure 5:**
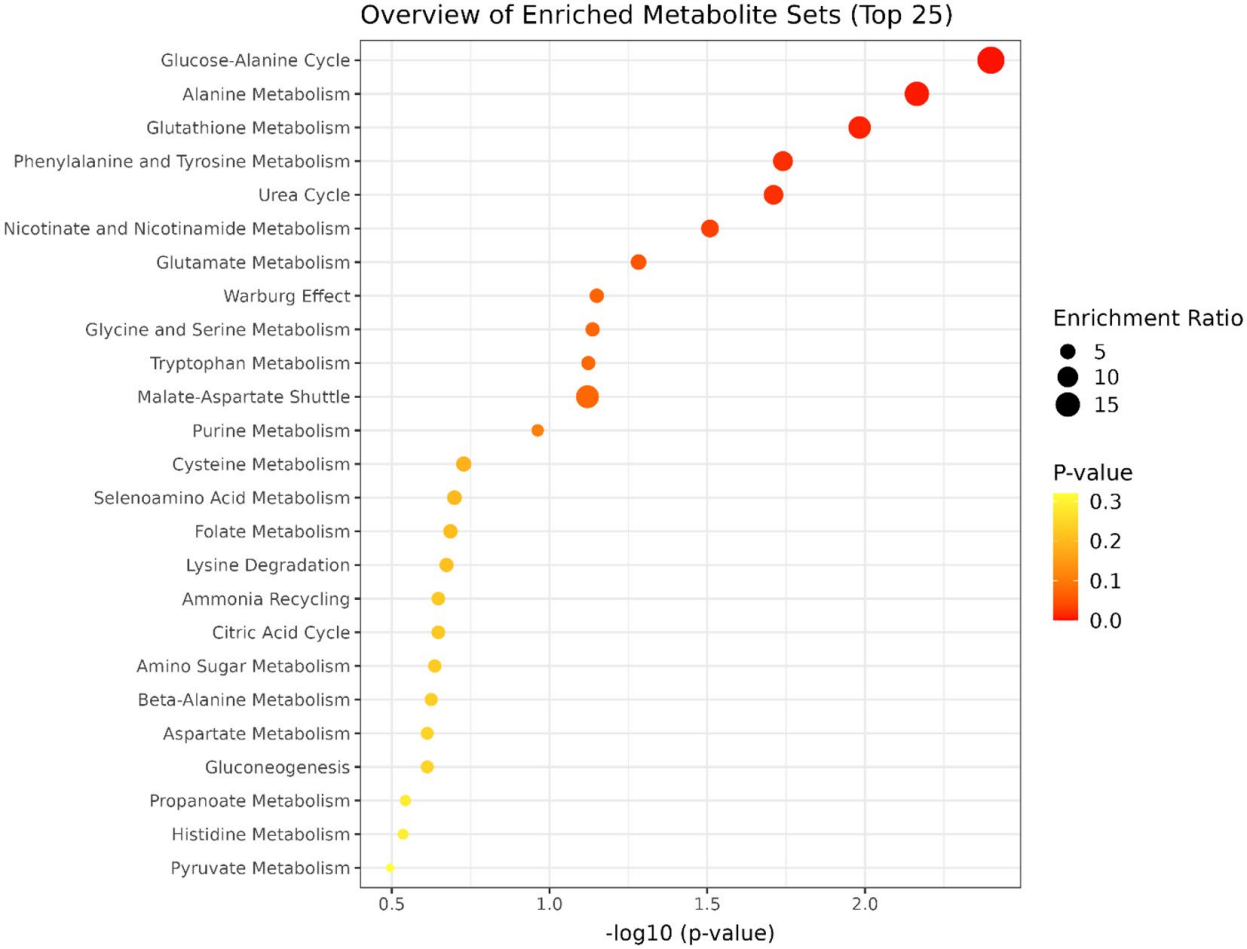
Quantitative enrichment analysis highlighting the metabolic pathways that were enriched in cluster creep compared with cluster control on day 220. The dots summarize the main metabolite sets identified in this analysis; dots are colored based on their P-values and relative size is based on enrichment the ratio.

**Figure 6:**
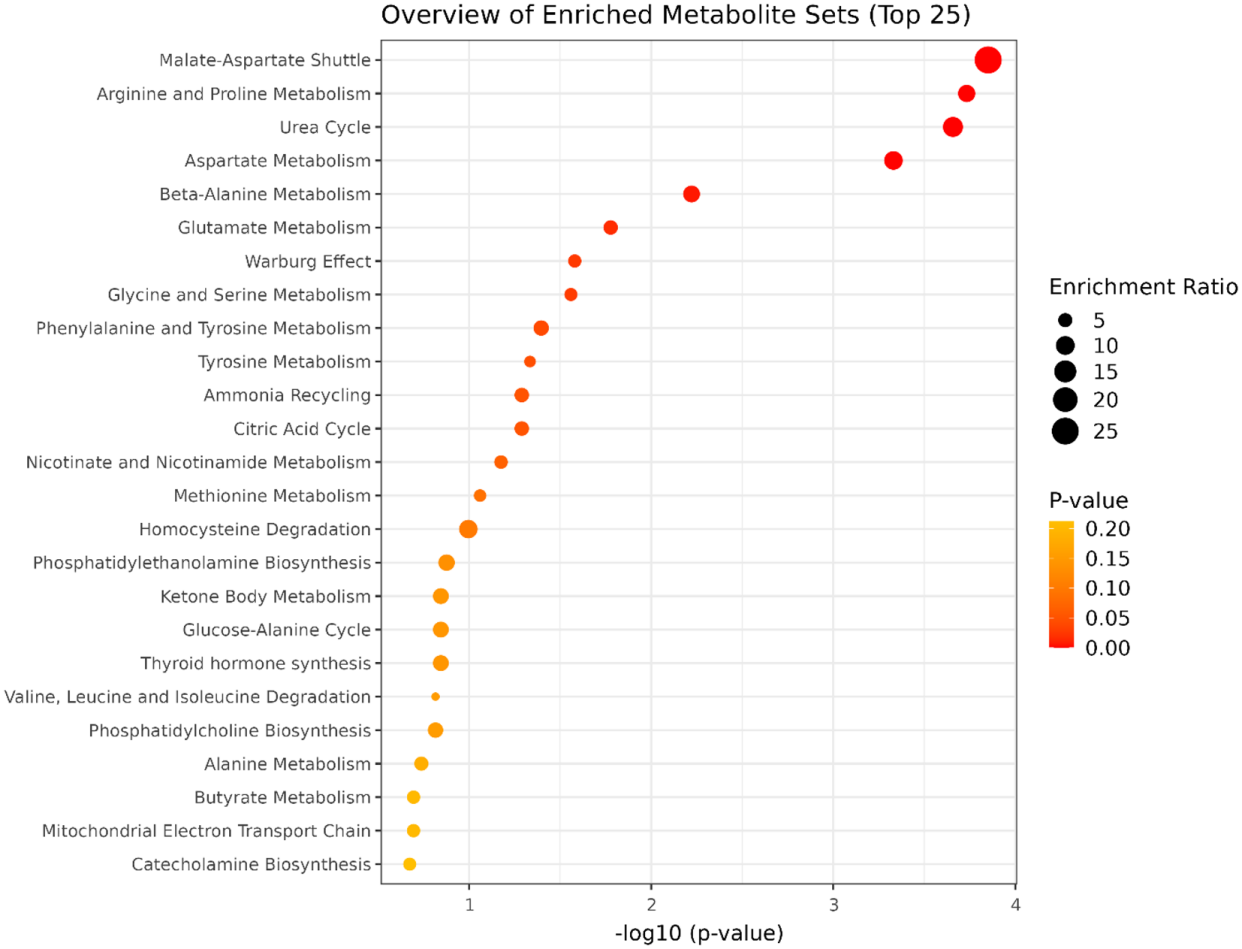
Quantitative enrichment analysis highlighting the metabolic pathways that were enriched in cluster creep compared with cluster control on day 360. The dots summarize the main metabolite sets identified in this analysis; dots are colored based on their P-values and relative size is based on the enrichment ratio.

The enrichment analysis of heifers'serum samples on Day 220 indicated five significant biological processes related to differentially expressed metabolites between Creep vs. Control groups (Fig. 5). The significant metabolic processes were the glucose-alanine cycle, alanine metabolism, glutathione metabolism, phenylalanine, and tyrosine metabolism, and the urea cycle. The list of enriched pathways within each metabolite involved is presented in Supplementary Table S2. In addition, eight significant biological processes were found on Day 360 (Fig. 6): Malate-aspartate shuttle, arginine and proline metabolism, urea cycle, aspartate metabolism, beta-alanine metabolism, glutamate metabolism, ammonia recycling and citric acid cycle. The list of enriched pathways within each metabolite involved is presented in Supplementary Table S3.

## Discussion

Based on the hypothesis about the potential impact of nutritional supplementation of nursing female calves on metabolic alterations affecting their future outcomes, our study provided significant insights into how the use of this strategy during early life can improve reproductive efficiency in heifers. Heifers supplemented with creep feeding showed higher BW and RFAT at weaning, as well as greater P/AI after the 1st service, resulting in a greater cumulative pregnancy rate at the end of their first breeding season, at 13–14 months old. These findings contribute to a better understanding of the intricate relationship between the metabolic imprinting promoted by early-life nutrition and the subsequent physiological responses in heifers. The creep feeding supplementation impacted the productive performance of the female calves at weaning, increasing their BW and fat deposition. Supporting these results, several studies reported positive effects of pre-weaning supplementation on productive aspects, such as BW^22,24,39^, ADG^23,24,40^, and RFAT^41,42^. Reis et al.^40^ reported the upregulation of genes associated with adipogenesis in calves receiving creep feeding, evidencing the pivotal role of fat tissue deposition in the future reproductive success of heifers^7,43^.

Aiming to better understand the metabolic alterations promoted by the nutritional treatment, a pathway analysis was performed, revealing significant modulation of the glucose-alanine cycle and alanine metabolism. Alanine, a non-essential amino acid abundant in both muscle and liver tissues of cattle, serves as a precursor for glucose synthesis^44^, particularly under low glucose where it becomes the primary substrate for hepatic gluconeogenesis^30,45^. Furthermore, alanine's involvement in glutathione metabolism is noteworthy. Glutathione, crucial for antioxidant defense, nutrient metabolism, and cellular regulation, relies on adequate protein intake to maintain homeostasis^46^. Studies in both animals and humans have demonstrated the importance of sufficient protein intake in preserving glutathione levels^47^. The modulation of glutathione metabolism, particularly during the weaning process, could significantly impact antioxidant capacity, cellular processes, and overall oxidative balance, underscoring the importance of maintaining adequate protein intake in calves.

Phenylalanine and tyrosine, both essential amino acids, have important roles in various biological processes, including the synthesis of proteins, neurotransmitters, and hormones in pregnant humans^48^. Recent research with calves experiencing faster growth rates have higher availability of amino acids, such as valine, leucine, isoleucine, phenylalanine, and tyrosine^49^, highlighting their importance in promoting growth and development. During the early stages of life, calves require large amounts of protein intake to fuel their rapid growth. These proteins are subsequently broken down into amino acids, which serve in various physiological processes, such as protein synthesis, enzyme function, hormone regulation, immune function, and energy production^50,51^. However, this breakdown process, along with microbiota fermentation in the rumen produces ammonia as a byproduct. When accumulated, ammonia can become toxic to the body. This underscores the importance the urea cycle plays in regulating nitrogen balance, a vital aspect of metabolic homeostasis^52^. In the context of this study, the Creep group received a supplement with feed additives known to improve digestibility, optimize nutrient utilization, and modulate the ruminal fermentative process. These enhancements in early-life ruminal metabolism could lead to improved feeding behavior and accelerated calf growth rates. Such changes hold significant implications for long-term productivity outcomes in beef cattle systems^34,53,54^. Reviewed by Lech et al.^55^, the metabolic effects of different feeding regimes in Holstein–Friesian cows post-calving were evaluated, revealing distinct metabolic adaptations in grass-fed versus grain-fed cows. Grass-fed cows had a ketogenic metabolism characterized by lower deuterium content in fatty acid products, while grain-fed cows exhibit elevated deuterium levels due to carbohydrate and protein-rich diets. The metabolic adaptations observed in grain-fed cows, including increased branched-chain amino acids and odd-chain fatty acids, may contribute to conditions like heart failure, diabetes, and obesity. The findings underscore the importance of understanding the metabolic consequences of different feeding practices, particularly regarding deuterium content, which may have implications for disease prevention and management.

After weaning, heifers from both experimental treatments were raised under the same nutritional and environmental conditions until the conclusion of the experiment. Notably, there were no significant differences in productivity parameters, such as BW, BFAT, and BCS at Days 360 and 408. The equivalence in BW could be attributed to a lower ADG during the post-weaning period, which can be explained by the challenges posed by a dry season with limited forage availability^34^. Furthermore, no differences were found between treatments when uterine and follicular diameter were evaluated. In addition, in this study, the nutritional supplementation did not anticipate puberty in supplemented heifers compared with the Control group. This finding corroborates a previous study with Bos indicus heifers, in which the treatment with creep feeding had no impact on the timing of puberty^40,56^. Surprisingly, in heifers supplemented with creep feeding the P/AI after 1st service was 28.9% (10.4 percentage points) higher than in the Control group (46.8 vs. 36.4%). This result prompts consideration of various underlying processes that might contribute to this notable increase in pregnancy rates, such as metabolic imprinting. It is reasonable to infer that additional nutrients provided through creep feeding could trigger metabolic changes during crucial developmental windows, leading to lasting effects on reproductive function^15^. This hypothesis is consistent with the idea that nutritional manipulation during early life could modulate metabolic pathways, potentially influencing later reproductive outcomes. This metabolic programming might impact hormonal regulation, oocyte quality, and embryo development, thereby optimizing fertility^11,29,57^.

Distinct metabolic variations among the animals emerged on day 360, highlighting pathways related to energy and glucose utilization. This metabolic insight is particularly crucial as the primary fuel source used by the central nervous system which plays a major role in the release of GnRH^58^. Notably, a prominent metabolic pathway that warrants attention is the malate-aspartate shuttle. This biochemical process is pivotal in cellular energy metabolism, particularly in the intricate transfer of reducing equivalents (electrons) between the cytosol and mitochondria^59^. This pathway involves a sequence of conversions involving malate's transformation to oxaloacetate within the mitochondria. The resulting oxaloacetate is then transported to the cytosol, where it’s converted into aspartate. This aspartate, in turn, is transported back into the mitochondria, establishing a dynamic cycle^60^. Notably, reproduction is a complex and multifactorial trait that hinges on various biological processes, including energy metabolism^61^. Within this context, the malate-aspartate shuttle is essential to produce the energy needed for oocyte maturation and fertilization^59^. Mitchell et al.^62^ examined the role of the malate-aspartate shuttle in nutrient metabolism pathways in developing mouse blastocysts. The findings indicated that impaired glucose metabolism via the malate-aspartate shuttle in blastocyst affected their ability to implant and form a pregnancy. Also, reduced fetal weight was observed, which may be attributed to placental development and function alterations. The observed variations in the citric acid cycle, a fundamental pathway crucial for both energy production and the synthesis of essential cellular molecules^63^, likely play a significant role in shaping the differences in reproductive performance between the Control and Creep groups. The citric acid cycle, also known as the Krebs cycle, is central to cellular metabolism, providing key intermediates for various biochemical processes. The nuanced changes in this pathway could have cascading effects on the energetic and biosynthetic processes essential for successful reproduction, thereby influencing the divergent reproductive outcomes observed in the two groups.

Arginine and proline metabolism play integral roles in several physiological processes associated with cattle fertility^45^. Arginine is an essential part of the urea cycle, and it is involved in the synthesis of polyamines and serves as a precursor to nitric oxide^50^. Nitric oxide and polyamines are important for implantation, embryonic survival, and development, as well as trophoblast outgrowth and cell migration^64^. According to Santana et al.^65^, adding L-arginine to the culture of bovine embryos promoted better preimplantation development. Also, it has been reported that dietary supplementation with arginine to sheep between the onset of estrus and day 25 after breeding enhanced embryonic and fetal survival during early pregnancy^66^. Proline, a non-essential amino acid, is actively involved in protein synthesis, particularly in the formation of structural proteins like collagen. It also contributes to nutritional processes, engages in antioxidative reactions and immune responses^50^. The activation of Proline metabolism in the present study suggests that supplemented heifers had higher concentrations of these amino acids. This is particularly relevant when considering previous research findings, where maternal undernutrition in pregnant ewes^67^ and pigs^68^ for both the first half of gestation and throughout gestation resulted in reduced concentrations of proline and polyamines in maternal plasma and the conceptus, as well as intrauterine growth retardation. Our results, indicating an enhancement in these amino acids, offer a counterpoint and imply positive effects on metabolic processes crucial for fetal development.

As previously discussed, alterations in the urea cycle were observed on day 220 and also on day 360, underscoring its critical role in ruminal function. Additionally, it's noteworthy that urea permeates into various body fluids, including blood and milk, achieving equilibrium in different body compartments, including the reproductive tissues^69^. This highlights the far-reaching influence of the urea cycle beyond the rumen, extending into vital physiological processes^70^. The activation of aspartate metabolism observed in the present study, particularly its role in the urea cycle, aligns with previous findings demonstrating its significance in promoting cellular processes essential for reproduction. Aspartate, in combination with ammonia forms arginosuccinate, a precursor for arginine and fumarate^71^. In cattle, aspartate is an important precursor for synthesizing other molecules, such as nucleotides and pyrimidines, which are critical for cell growth and division and the synthesis of new cells. Drawing parallels with an intriguing study^59^, our results suggest that the heightened concentrations of aspartate could play a pivotal role in supporting reproductive success.

Heifers from the Creep group showed differences in beta-alanine metabolism at day 360. The primary function of beta-alanine metabolism in cattle is the synthesis of carnosine, which has been shown to possess antioxidant and anti-inflammatory properties and is involved in various physiological processes such as muscle function and acid–base regulation^72,73^. Another vital antioxidant involved was glutathione, synthesized by glutamate metabolism. Glutamate, an amino acid found abundantly in female reproductive fluids^73^, promotes the synthesis and degradation of proteins and participates in cellular signal transduction, gene expression regulation, and metabolic cascades^74^. Glutamate has been shown to enhance the productivity of pubertal rams and nulliparous ewes^75^ and is closely associated with protein metabolism, acting as an anabolic mediator that decreases protein catabolism and promotes an increase in muscle growth rate^76^.

In summary, this study demonstrates the positive impact of creep feeding supplementation on the weaning performance of female calves and unveils notable alterations in metabolic pathways related to energy production, nutrient metabolism, antioxidant defense, and ruminal function. Although heifers did not exhibit differences in productive performance and reproductive status during the TAI protocol, those subjected to creep feeding had a higher P/AI after 1st service and cumulative P/AI. Additionally, the study identified significant changes in metabolic pathways during this critical phase involving energy production, protein synthesis, muscle function, antioxidant process, cellular growth, neurotransmitter synthesis, and ruminal function. These findings suggest that creep feeding can be a valuable management practice for inducing metabolic imprinting, ultimately enhancing both calf performance and reproductive efficiency in young Nelore heifers. Implications of these findings include informing livestock management strategies to optimize calf health and productivity. Further steps may involve longitudinal studies to assess the long-term effects of creep feeding on calf growth, health, and reproductive performance, as well as exploring potential mechanisms underlying the observed metabolic adaptations.

### Supplementary Information

Supplementary Information.

## Data Availability

All data generated or analyzed during this study are included in this published article and its supplementary information files.
